# A diagnostic instrument to help field graders evaluate active trachoma

**DOI:** 10.1080/09286586.2018.1500616

**Published:** 2018-08-01

**Authors:** Anthony W. Solomon, Richard T. Le Mesurier, William J. Williams

**Affiliations:** a Department of Control of Neglected Tropical Diseases, World Health Organization, Geneva, Switzerland; b Medical Division, The Fred Hollows Foundation, Melbourne, Australia; c School of Medicine, University of St Andrews, St Andrews, UK; d Arclight Medical, Liverpool, UK

**Keywords:** Diagnosis, epidemiology, trachoma

## Abstract

The SAFE strategy (Surgery for trichiasis, mass treatment with Antibiotics to clear ocular *Chlamydia trachomatis* infection, and Facial cleanliness and Environmental improvement to reduce transmission) is being used to eliminate trachoma as a public health problem. Decisions on whether or not to implement the A, F, and E components of SAFE are made on the basis of the prevalence of trachomatous inflammation—follicular (TF) in 1–9-year-olds. TF has a precise definition: at least five follicles, each of which is at least 0.5-mm diameter, in the central part of the upper tarsal conjunctiva. Determining whether a follicle has a diameter ≥0.5mm is difficult using magnifying loupes alone. We have developed an ultra-low-cost solution: a follicle size guide that takes the form of a durable printed adhesive sticker which can be fixed to graders’ thumb nails for direct size comparison. This tool will be made available to health ministries free of charge. It is anticipated to simplify grader training, increase grader trainee pass rates, and prevent in-service diagnostic drift after training is complete.

Since 1998, trachoma has been formally targeted for elimination as a public health problem worldwide.^1^ The need or otherwise for public health-level interventions against trachoma, and the success or otherwise of those interventions in achieving elimination prevalence targets, are determined through the use of population-based prevalence surveys. The throughput and reproducibility of such surveys increased dramatically with the implementation of the Global Trachoma Mapping Project (GTMP), which ran from December 2012 to January 2016.^2,3^ Its quality control and quality assurance mechanisms^4^ were carried over to and reinforced within the systems of its successor, Tropical Data.^5^

Despite these efforts to ensure quality, a persistent Achilles heel for trachoma surveys is their reliance on assessment of the presence or absence of signs of disease.^6^ These signs are defined in ways that appear precise, but the application of the definitions has—unavoidably to date—been somewhat subjectively applied, despite the best efforts of those using them. In particular, the key index for decision-making on implementation of the A (antibiotics), F (facial cleanliness), and E (environmental improvement) components of the WHO-recommended “SAFE strategy”^7^ for trachoma elimination purposes is the prevalence in 1–9-year-olds of the sign “trachomatous inflammation—follicular” (TF). TF is a sign of active (inflammatory) trachoma from the WHO-simplified trachoma grading system defined as “*the presence of five or more follicles in* [the central part of] *the upper tarsal conjunctiva*,” where to be counted, “*follicles must be at least 0.5mm in diameter*.”^8^ In order to train and certify the 611 GTMP graders that completed mapping in 1546 districts of 29 countries, health ministries recruited ophthalmic nurses, many of whom were already experienced; used the rigorous GTMP training scheme; and tested proficiency in TF diagnosis using formal inter-grader agreement exercises in which groups of 50 real children acted as the examination subjects.^9^ Some 20–30% of candidate graders failed.^10^

As programes progress toward elimination endpoints, several thousand more district-level population-based surveys will be needed.

Apart from the inherent difficulty in consistently determining the “central part of the conjunctiva” as the area to be examined (Figure 1), there is an obvious challenge in ensuring that field graders are clear in their minds as to how big 0.5 mm actually is (Figure 2), a requirement that is not made any easier by the fact that the features of interest are viewed (a) through 2.5× magnifying loupes,^3^ and (b) a at distance that varies depending on examination conditions and the response of the subject to the experience of being examined. Though efforts have been made to develop image capture systems for centralized grading by experts, technical obstacles remain.^11–14^

We have developed a simple, ultra-low-cost solution to this problem, in the form of a follicle size guide printed on small plastic stickers (Figure 3) that are easily fixed to graders’ thumbnails. Once firmly attached to a clean, grease-free nail, they are washable with alcohol gel or water and soap. With minimal care, they remain in place all day despite repeated cleaning. Informal tests show they can in fact stay on for more than 5 days.

Whilst an oval sticker might better reinforce the central-conjunctival-area concept, a more practical shape for nail application and reference is an orientation-free, 8.5-mm-diameter circle. The background color is an approximation of the usual color of an inflamed conjunctiva, printed at Pantone 171C (RGB: 255, 92, 57). The five white dots are each 0.5 mm in diameter with a slightly dithered edge. The dithering is a limitation of the printing process but is also actually helpful, in that it makes the dots appear more like real follicles. The polycarbonate sticker material is 0.125-mm thick, with a matte, low-reflection, easy wipe finish. The pressure adhesive is a 3M 467MP type that gives a low profile but a secure grab. Presentation is as 230 mm × 297 mm sheets, each of which incorporates 25 rows of 20 stickers.

When examining the conjunctiva for trachoma, after everting the eyelid, the grader’s thumb is generally used to maintain the eyelid in the everted position by holding the eyelashes against the superior orbital margin. This means that a follicle size guide affixed to the thumbnail lies in nearly the same optical plane as the conjunctiva, allowing easy comparison of the size of the dots and any follicles (Figure 4). Wearing a follicle size guide on each thumb is important, since the grader’s left thumb is used to hold the subject’s right eyelid in the everted position, and the grader’s right thumb is used to hold the subject’s left eyelid.^9^

Accuracy and repeatability of diagnostic methods are issues for many areas of clinical medicine, epidemiology, and medical research.^15–17^ The GTMP painstakingly constructed systems to maximize the reliability of data amassed through the surveys that they supported,^4^ including a rigorous training cascade to produce certified trachoma graders for fieldwork.^3^ This latest addition to the system will be made available to health ministries at no cost, and is expected to further enhance diagnostic accuracy, justifying even greater confidence in the prevalence estimates generated by national programs^18–22^ in their path toward elimination of trachoma as a public health problem.

## Figures and Tables

**Figure 1:**
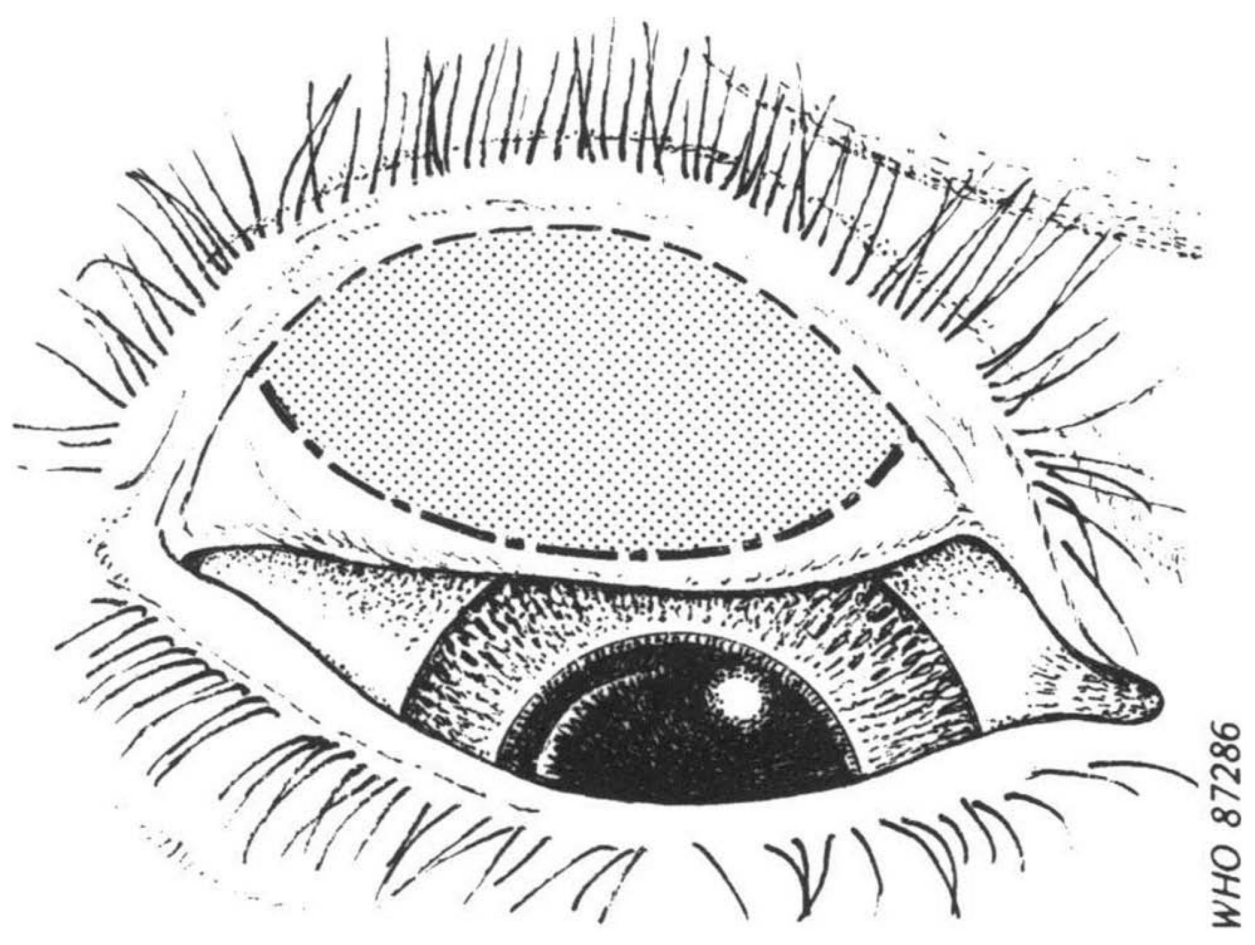
Sketch of everted upper eyelid, showing the area (shaded) of the tarsal conjunctiva to be examined for assessment of trachomatous inflammation—follicular^8^ (© World Health Organization, reproduced with permission).

**Figure 2:**
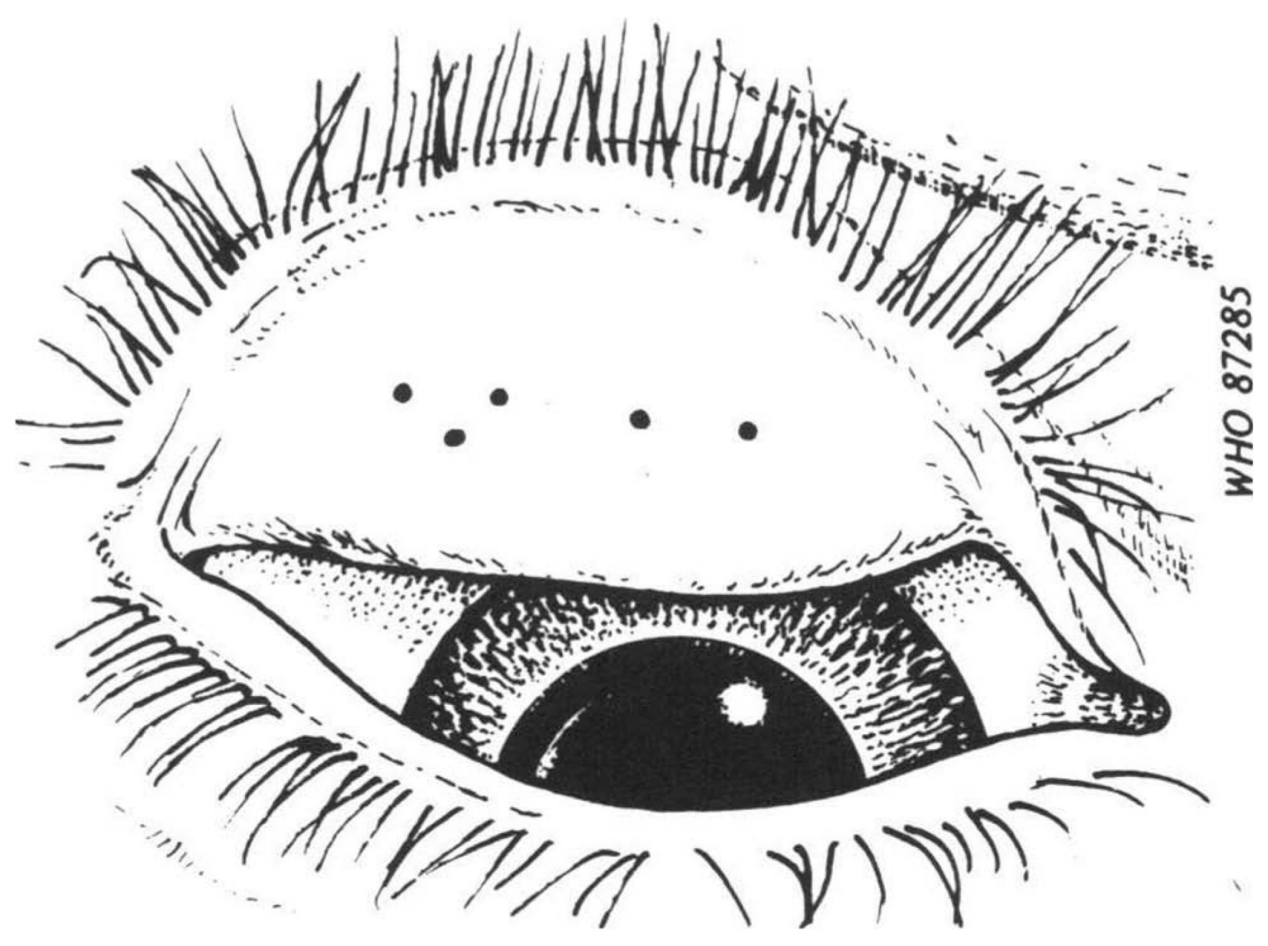
Sketch of an everted upper eyelid with five central conjunctival follicles of 0.5mm diameter^8^ (© World Health Organization, reproduced with permission).

**Figure 3:**
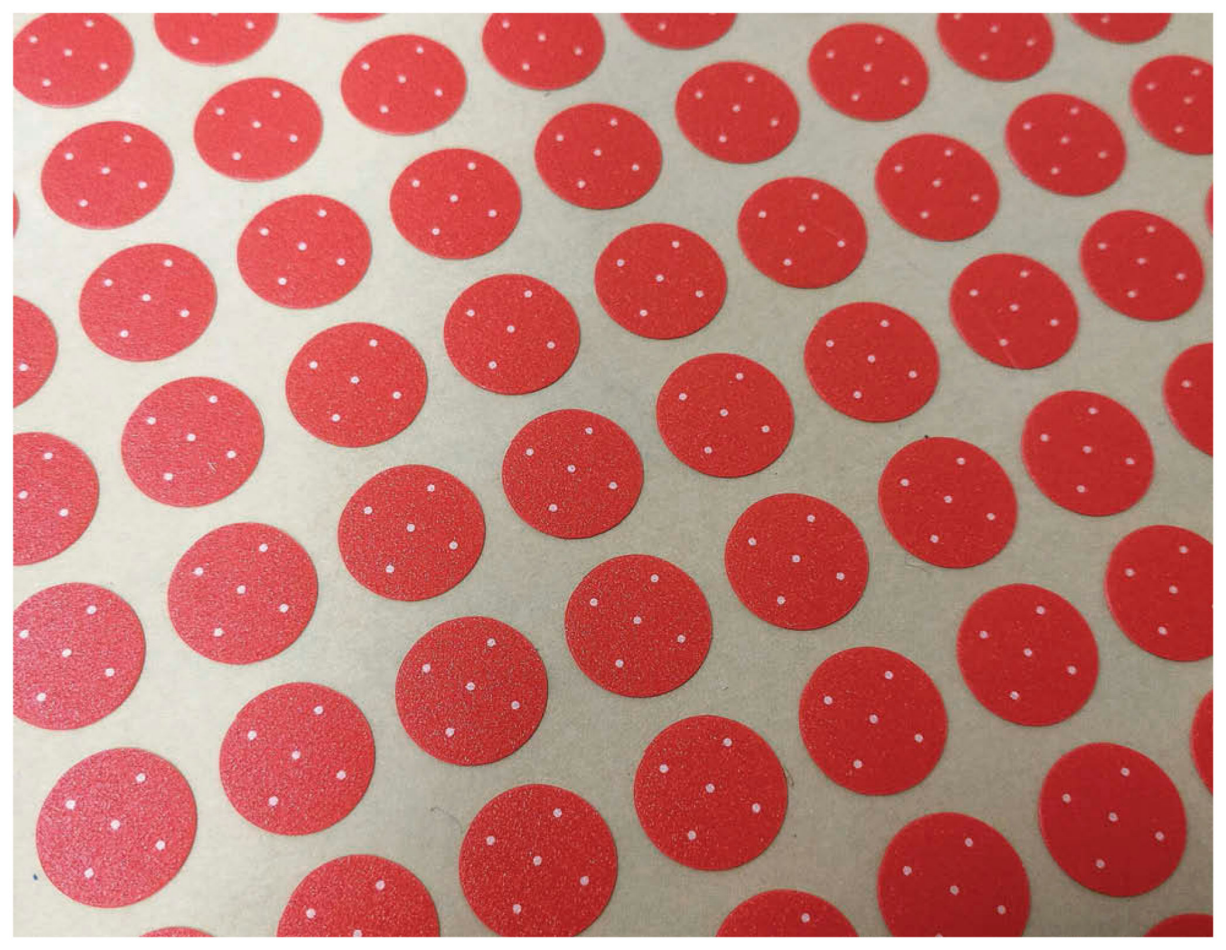
A sheet of follicle size guides. Each sticker bears five dots, each of diameter 0.5 mm.

**Figure 4:**
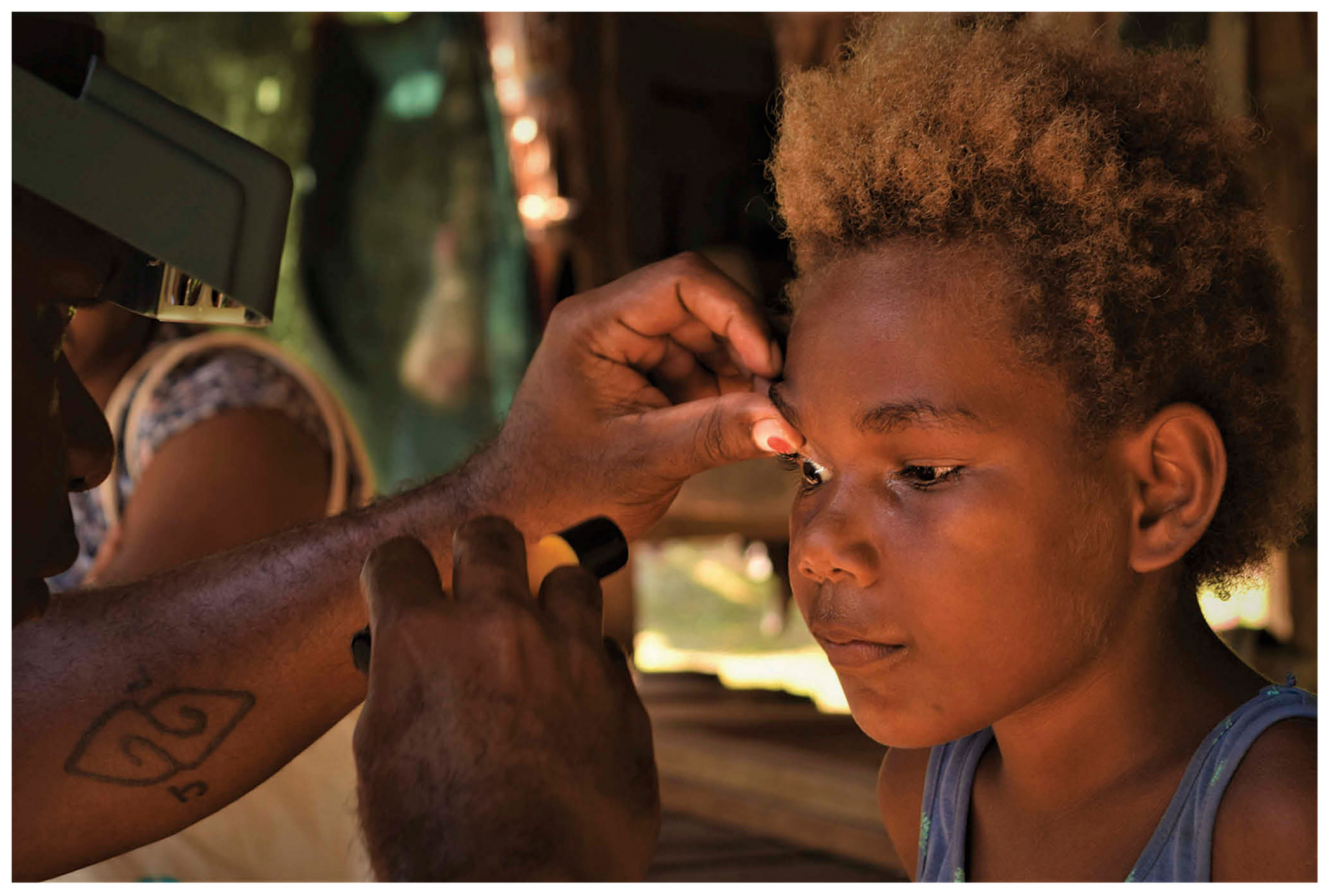
A follicle size guide in use. (Photo: Shea Flynn/RTI International/Tropical Data, reproduced with permission).
